# Commentary: Amplification and Suppression of Distinct Brainwide Activity Patterns by Catecholamines

**DOI:** 10.3389/fnbeh.2019.00217

**Published:** 2019-09-18

**Authors:** Vicente Medel, Joaquín Valdés, Samy Castro, Tomás Ossandón, Gonzalo Boncompte

**Affiliations:** ^1^Centro Interdisciplinario de Neurociencias, Pontificia Universidad Católica de Chile, Santiago, Chile; ^2^Neurodynamics of Cognition Laboratory, Departamento de Psiquiatría, Facultad de Medicina, Pontificia Universidad Católica de Chile, Santiago, Chile; ^3^Neural Dynamics Laboratory, Centro Interdisciplinario de Neurociencia de Valparaíso, Universidad de Valparaíso, Valparaíso, Chile; ^4^Programa de Doctorado en Ciencias, Mención Neurociencia, Universidad de Valparaíso, Valparaíso, Chile

**Keywords:** locus coeruleus (LC), norepinephrine (NE), catecholaminergic receptors, atomoxetine (ATX), fMRI, iEEG, pupil diameter

Brain states like sleep and vigilance, as well as fluctuating levels of arousal and attention, are characterized by diverse patterns of brain activity. These global dynamics are strongly driven by the activity of catecholaminergic neuromodulatory systems (Sara and Bouret, 2012; Reimer et al., 2014; van den Brink et al., 2016). Specifically, norepinephrine (NE) levels have been shown to be coupled to brain states (Eschenko et al., 2011; McGinley et al., 2015). The cortical influence of NE comes from neurons originating in the locus coeruleus (LC) which has widespread projections to the forebrain and has been assumed to have a uniform impact on brain activity. However, neuromodulatory effects vary in part because of the heterogeneous cortical distribution of NE synaptic receptors (Zilles and Amunts, 2009) which suggests that cortical modulation of NE is more complex than previously thought (Totah et al., 2018).

Using fMRI and pharmacological intervention, van den Brink et al. (2018) sought to determine whether NE modulation on brainwide interactions occurred in a spatially distributed manner depending on receptor genes. For this, they analyze resting-state fMRI functional connectivity (FC) in healthy subjects under both placebo condition and a pharmacological increase of NE levels by a single dose of atomoxetine (ATX), an inhibitor of the NE transporter. They use a previously proposed approach (Donner et al., 2013) to decompose the FC matrices into spatial modes of brain organization that capture the heterogeneous atomoxetine-induced effects over intrinsic brain variations.

To compare the spatial modes with well-known brain characteristics, the authors correlate these spatial modes with canonical resting-state FC networks (Smith et al., 2009). Interestingly, the ATX spatial mode correlates with the right frontoparietal network (FPN) while the placebo spatial mode correlates with the left FPN and the default-mode network, which has important roles in cognition. This is of special interest, considering that these networks are obtained from the resting-state, which suggests that slow spontaneous fluctuations are modulated by NE even in the absence of task. Indeed, the authors interpret that ATX might induce a shift toward a goal-oriented stimulus processing brain state. Considering the computational evidence that resting-state fluctuations may arise from slow fluctuation of ionic concentrations (Krishnan et al., 2018), van den Brink et al. (2018) results experimentally supports the understanding of catecholaminergic modulation as a spatially heterogeneous gain function of biophysical dynamics (Shine et al., 2018).

If the above were true, a strong coupling between the spatial modes and the localization of NE receptors would be expected. Indeed, using the receptor's transcriptional maps from the Allen Brain Institute (Hawrylycz et al., 2015), the authors show that the heterogeneous spatial modes are partially explained by the spatial heterogeneity of NE receptors. Specifically, the distribution of the spatial modes significantly correlates with the localization of β NE receptors and with α1 NE receptors, but not with α2 NE receptors or NMDA receptors. This is of special interest because α2 shows higher affinity to NE than α1 receptors (Berridge and Spencer, 2016). Both of these receptors are known to be cognitively important but in different ways. α2 activation has been linked to enhanced working memory capacity, while α1 is related to high arousal situations and impaired working memory while promoting attention flexibility (Berridge and Spencer, 2016). This is in line with the adaptive gain theory proposed by Aston-Jones and Cohen (2005), which links LC-NE activity with cognitive performance. Interestingly, as the authors note, there is a significant expression of NE receptors in subcortical areas, including α2 NE autoreceptors in the LC, which should be taken into account to describe these complex phenomena. However, and perhaps more importantly, recent evidence has shown that ATX has opposite effects in network integration in resting state compared to cognitive tasks, which supports a state-dependent modulation of brain connectivity by catecholamines (Shine et al., 2019).

Humans interact with the dynamic nature of the world with a high temporal resolution. Placing van den Brink et al. (2018) findings into the perspective of spontaneous fluctuations in cognition, it appears as highly relevant to characterize the dynamic shaping of brain activity by neuromodulators on a finer temporal scale using electrophysiology (e.g., McGinley et al., 2015). In this line, Pfeffer et al. (2018) found that a single dose of ATX shapes an aperiodic measure of the field potential during perception of ambiguous visual stimuli. This is consistent with evidence that proposes aperiodic measures such as the level of background neural activity (Voytek and Knight, 2015) as physiological markers of network dynamics. Interestingly, the aperiodic activity has been shown to highly correlate with spiking activity (Manning et al., 2009), and is a good electrophysiological correlate of the BOLD signal (Wen and Liu, 2016), emerging as a candidate to link micro and macro scale in the study of neuromodulation of brain activity. Thus, it is tempting to test if the spatial modes revealed by fMRI are spatially coincident with electrophysiological field potential patterns, such as aperiodic broadband, as previous studies have done (Ossandón et al., 2011).

The results presented by van den Brink et al. (2018) extend our understanding of the fine-grained spatial architecture of brain activity and its reshaping by ATX. Although pharmacological interventions studies contribute to elucidate the catecholaminergic effects on cortical states, they fail to describe its naturally dynamic fluctuations. Given the well-established role of the LC in driving cortical states and pupil diameter (Aston-Jones and Cohen, 2005; Yüzgeç et al., 2018), pupillometry appears as an excellent candidate to relate endogenous time-varying NE levels with brain states (Reimer et al., 2014; Wainstein et al., 2017).

van den Brink et al. (2018) contribute to the challenge of linking macro scale brain organization with low-level characteristics of neurotransmitter receptors. Extending these important results using higher temporal resolution methods, as intracranial EEG, and adding in parallel pupillometry would give a broader understanding of how neuromodulators spatially interact with brain state fluctuations and cognition. This could potentiate future research to understand the multiscale functional dynamics underlying several neuromodulator-related psychiatric disorders as well as to pave the path to design targeted therapeutic strategies.

## Author Contributions

All authors listed have made a substantial, direct and intellectual contribution to the work, and approved it for publication.

### Conflict of Interest Statement

The authors declare that the research was conducted in the absence of any commercial or financial relationships that could be construed as a potential conflict of interest.
